# Inhibition of Apoptosis and NF-κB Activation by Vaccinia Protein N1 Occur via Distinct Binding Surfaces and Make Different Contributions to Virulence

**DOI:** 10.1371/journal.ppat.1002430

**Published:** 2011-12-15

**Authors:** Carlos Maluquer de Motes, Samantha Cooray, Hongwei Ren, Gabriel M. F. Almeida, Kieran McGourty, Mohammad W. Bahar, David I. Stuart, Jonathan M. Grimes, Stephen C. Graham, Geoffrey L. Smith

**Affiliations:** 1 Department of Virology, Faculty of Medicine, Imperial College London, London, United Kingdom; 2 The Division of Structural Biology, Wellcome Trust Centre for Human Genetics, University of Oxford, Oxford, United Kingdom; 3 Science Division, Diamond Light Source, Didcot, United Kingdom; University of Alberta, Canada

## Abstract

Vaccinia virus (VACV) protein N1 is an intracellular virulence factor and belongs to a family of VACV B-cell lymphoma (Bcl)-2-like proteins whose members inhibit apoptosis or activation of pro-inflammatory transcription factors, such as interferon (IFN) regulatory factor-3 (IRF-3) and nuclear factor-κB (NF-κB). Unusually, N1 inhibits both apoptosis and NF-κB activation. To understand how N1 exerts these different functions, we have mutated residues in the Bcl-2-like surface groove and at the interface used to form N1 homodimers. Mutagenesis of the surface groove abolished only the N1 anti-apoptotic activity and protein crystallography showed these mutants differed from wild-type N1 only at the site of mutation. Conversely, mutagenesis of the dimer interface converted N1 to a monomer and affected only inhibition of NF-κB activation. Collectively, these data show that N1 inhibits pro-inflammatory and pro-apoptotic signalling using independent surfaces of the protein. To determine the relative contribution of each activity to virus virulence, mutant N1 alleles were introduced into a VACV strain lacking N1 and the virulence of these viruses was analysed after intradermal and intranasal inoculation in mice. In both models, VACV containing a mutant N1 unable to inhibit apoptosis had similar virulence to wild-type virus, whereas VACV containing a mutant N1 impaired for NF-κB inhibition induced an attenuated infection similar to that of the N1-deleted virus. This indicates that anti-apoptotic activity of N1 does not drive virulence in these *in vivo* models, and highlights the importance of pro-inflammatory signalling in the immune response against viral infections.

## Author Summary

Viruses have multiple strategies to escape the host immune system. These include proteins to inhibit cellular signalling pathways promoting an inflammatory response, and others that prevent programmed cell death (apoptosis), allowing completion of the virus replication cycle. This paper concerns the vaccinia virus (VACV) protein N1, which forms homodimers and blocks activation of both apoptosis and the pro-inflammatory NF-κB transcription factor. By introducing mutations in N1, we demonstrate that these functions are mediated by different surfaces of the protein. Biochemical and structural analysis of these mutants demonstrates that the anti-apoptotic activity of N1 relies on a hydrophobic groove on the surface of the protein and that the anti-NF-κB activity requires an intact dimer interface. Recombinant VACVs expressing the mutant N1 proteins were made to investigate the contributions of the different properties of N1 to virulence. The results showed that the anti-NF-κB activity of N1, rather than the N1-mediated inhibition of apoptosis, is the major contributor to virulence. This underlines the central role of pro-inflammatory signalling in the host immune response against viral infections.

## Introduction

Viral infections are sensed by pattern recognition receptors that activate the innate immune system which precedes the development of an adaptive immune response. The strength of this initial innate immune activation determines the quality of the acquired immune responses and, consequently, the progression and outcome of the infection. Vaccinia virus (VACV), the smallpox vaccine, has a large double-stranded DNA genome of about 190 kb. Approximately half of the genes are non-essential for virus replication and many of these modulate the innate immune response [1], [2]. VACV protein N1 is one of these immune modulators. N1 is a dimeric cytosolic protein expressed early during infection [3] that contributes to viral virulence [3]–[5]. It belongs to a family of VACV proteins displaying a B cell lymphoma (Bcl)-2-like structural fold whose members inhibit apoptosis or activation of pro-inflammatory transcription factors [6]–[12]. N1, however, is unusual in that it inhibits both pro-apoptotic and pro-inflammatory signalling [7], [8], [13].

Pro-inflammatory signalling in epithelial cells is controlled by transcription factors such as IRF-3 and NF-κB. The NF-κB complex is a major regulator of the host antiviral innate immunity and consists of a family of dimeric transcription factors retained in the cytoplasm of resting cells by association with NF-κB inhibitory proteins (IκB) [14], [15]. Binding of cytokines interleukin-1 (IL-1) or tumour necrosis factor-α (TNFα) to their receptors or engagement of Toll-like receptors (TLRs) by their ligands leads to activation of signalling pathways that converge at the IκB kinase (IKK) complex. Once activated, the IKK complex then phosphorylates IκB causing its ubiquitination and degradation by the proteasome. Consequently, NF-κB is released and translocated into the nucleus where it induces transcription of NF-κB-dependent genes. Activation of NF-κB via the IL-1 receptor and TLRs requires the recruitment of the adaptor proteins myeloid differentiation factor 88 (MyD88) and TNF-receptor-associated factor 6 (TRAF6), whereas activation via the TNF receptor requires the kinase receptor interacting protein (RIP) and TRAF2 [14], [16]. N1 was reported to prevent activation of the transcription factors NF-κB and IRF-3 deriving from a variety of stimuli by targeting several components of the IKK and the TANK-binding kinase 1 (TBK1) complexes [13]. Other studies have also shown inhibition of NF-κB signalling by N1, but did not find interactions between N1 and components of the IKK or TBK1 complexes [7]–[9] and consequently the mechanism employed by N1 to prevent NF-κB activation remains unclear.

Apoptosis is an irreversible cascade of biochemical events orchestrated by caspase proteases that culminates in cell death and represents a potent mechanism that aids elimination of virus-infected cells [17]. Consequently, viruses have developed strategies to subvert apoptotic signalling and facilitate completion of their replication cycle [18], [19], including expression of Bcl-2 like proteins from Kaposi sarcoma-associated herpesvirus [20], [21], Epstein-Barr virus (BHRF1) [22]–[24], γ-herpesvirus 68 (vBcl-2) [25], [26], myxoma virus (M11) [27]–[30] and VACV (F1 and N1) [7], [8], [31], [32], [33], [34], [35]. A central feature of apoptosis is mitochondrial outer membrane permeabilization (MOMP) that leads to the release of cytochrome *c* to the cytosol and formation of the caspase activation platform known as the apoptosome. The Bcl-2 family of proteins regulates MOMP by complex protein-protein interactions between pro-apoptotic and anti-apoptotic members of the family [36], [37]. Anti-apoptotic proteins (Bcl-2, Bcl-xL, Bcl-w) contain four Bcl-2 homology motifs (BH1-4) and are generally integrated in the outer mitochondrial membrane (OMM). They prevent apoptosis by binding the BH3 motif of the pro-apoptotic Bcl-2 proteins. Pro-apoptotic Bcl-2 proteins are divided into the effector proteins (Bax, Bak), which homo-oligomerise and insert into the OMM to promote MOMP, and the BH3-only proteins, which either bind only the anti-apoptotic members (Bad, Noxa) or bind these as well as the pro-apoptotic effector proteins (Bid, Bim) [36], [38].

The crystal structure of N1 identified a groove similar to those of cellular anti-apoptotic Bcl-2 proteins [6], [8]. However, N1 lacks the C-terminal helix that regulates access to the binding groove and so is a constitutively ‘active’ anti-apoptotic protein [8]. Consistent with this, N1 interacts with endogenous Bad, Bax and Bid and protects cells from staurosporine (STS)-induced apoptosis [8]. Moreover, while a similar Bcl-2 fold is observed for other VACV proteins B14, A52 and K7 that are also inhibitors of NF-κB, neither a BH3-binding groove nor an anti-apoptotic activity are observed in these proteins [7], [39]. N1 is therefore unusual in its dual ability to modulate both apoptosis and inflammatory signalling.

In this study specific mutations were introduced into N1 that eliminate either its ability to prevent apoptosis or block NF-κB activation. N1 proteins that display only one of the two functions have been structurally and biophysically characterized, and recombinant VACV expressing these proteins were generated and their virulence was assessed in murine models. Data presented show that inhibition of apoptosis and NF-κB activation are mediated by different surfaces of the N1 protein and that mutation of N1 so that it no longer inhibits apoptosis did not affect virulence, whereas inhibition of pro-inflammatory signalling was important for virulence.

## Results

### Mutagenesis of vaccinia virus protein N1

**Figure 1:**
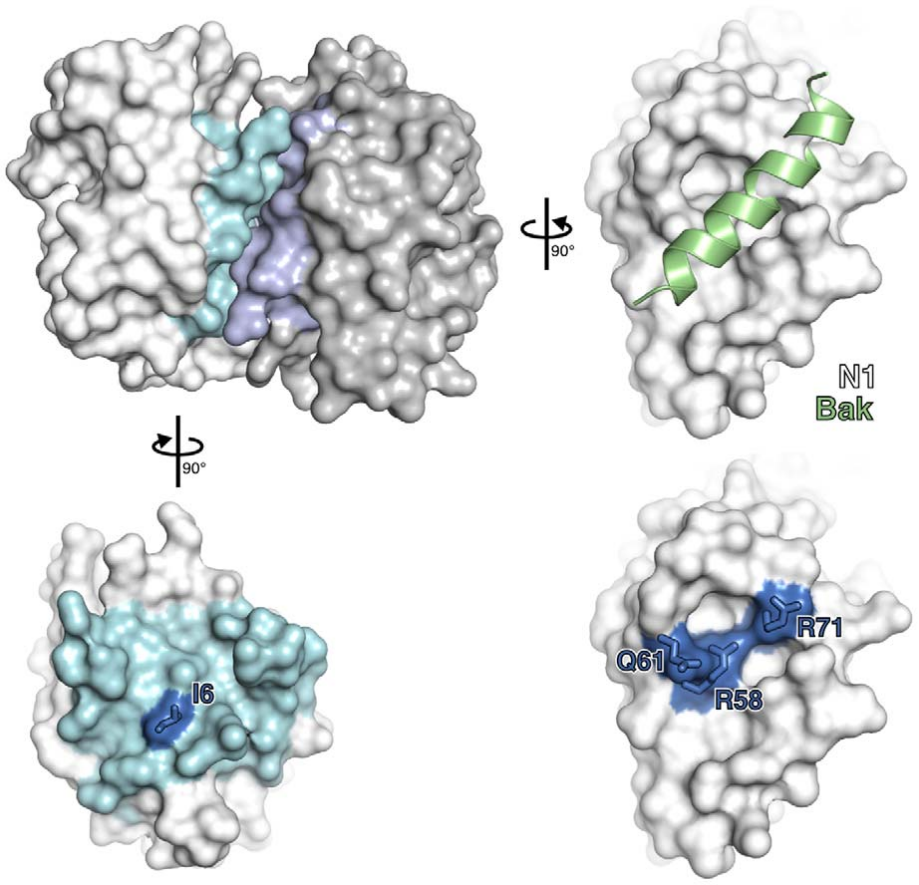
Mutagenesis of vaccinia virus protein N1. The structure of an N1 homodimer is shown as a molecular surface, the two molecules being coloured white and grey. Residues at the dimerisation interface of each molecule are highlighted in blue. A model of a BH3 peptide (light green helix) bound to N1, generated by superposition of N1 onto the M11:Bak BH3 peptide complex (PDB ID 2JBY), is shown (right). Mutations introduced in the BH3-peptide binding groove (lower right) and dimerisation interface (lower left) are shown in electric blue.

To understand how N1 interferes with pro-apoptotic and pro-inflammatory signalling, we introduced point mutations in N1 based on its crystal structure. N1 contains a surface groove predicted to bind BH3 peptides [6], [8]. By analogy with VACV proteins A52 and B14, which have the surface groove filled by bulky amino acid residues and do not inhibit apoptosis [7], we introduced the bulky residue tyrosine at positions R58, Q61 and R71 in an attempt to ‘fill’ the N1 groove and thereby exclude BH3-peptide binding (Figure 1). Mutations were also introduced at the hydrophobic surface that mediates formation of N1 dimers. The VACV Bcl-2-like proteins N1, B14 and A52 all form dimers via a common surface comprising alpha helices 1 and 6 (the ‘1–6 face’) [7], [8], [11], [39]. This same surface is also used by the VACV Bcl-2 family protein K7 to bind the host-cell protein DDX3 [7], [8], [39]. To determine whether dimer formation via this surface is important for N1 function, a hydrophobic to polar mutation (I6E) was introduced (Figure 1).

### Structures of wild-type and groove-filling mutant N1

**Figure 2:**
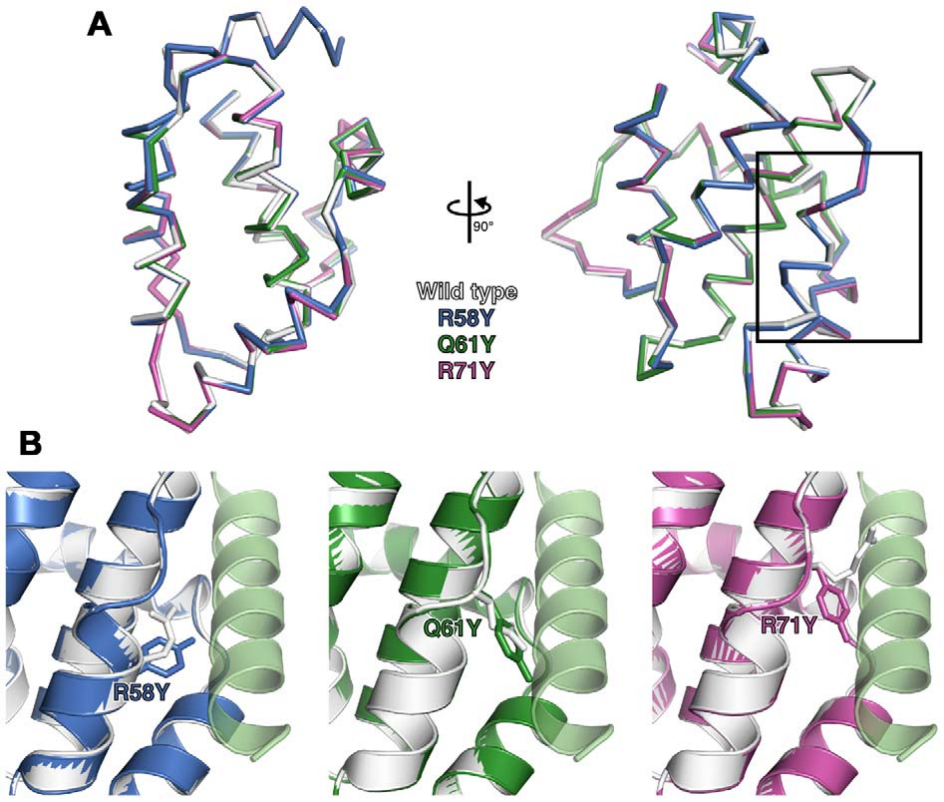
Structures of wild-type and groove-filling mutant N1. (**A**) The structures of wild-type and mutant N1 are shown as superposed Cα traces with two orthogonal views. The BH3 peptide binding groove enlarged in (B) is boxed. (**B**) Enlarged view of mutant N1 (coloured) superposed on wild-type N1 (white) with the position of the mutated residue shown as sticks. A model of a bound BH3 peptide is shown as in Figure 1.

To determine if the point mutations introduced in N1 generated unanticipated structural changes, R58Y, Q61Y and R71Y N1 were expressed in bacteria, purified to homogeneity and their structures determined at 3.0–3.1 Å resolution (Table S1). All three mutants crystallized in the same space group as wild-type N1 [8] and the overall structures of all three closely resembled the wild-type protein (0.21–0.39 Å r.m.s. deviation over 108 Cα atoms) (Figure 2A). In each case the point mutation could be readily identified and the mutated residues are shown in final refined structures in Figure 2B. For both the R58Y and Q61Y mutants, the tyrosine residue protruded into the groove in a manner likely to reduce BH3 peptide binding. However, in the R71Y mutant, the tyrosine lay along the bottom of the groove, not protruding upwards like the original arginine side chain and so the groove remained “open”. In all cases the mutated side chains were the only significant structural difference between wild-type and mutant N1. Unfortunately I6E N1 proved refractory to all crystallization attempts.

### Biochemical and biophysical characterization of N1 mutants

**Figure 3:**
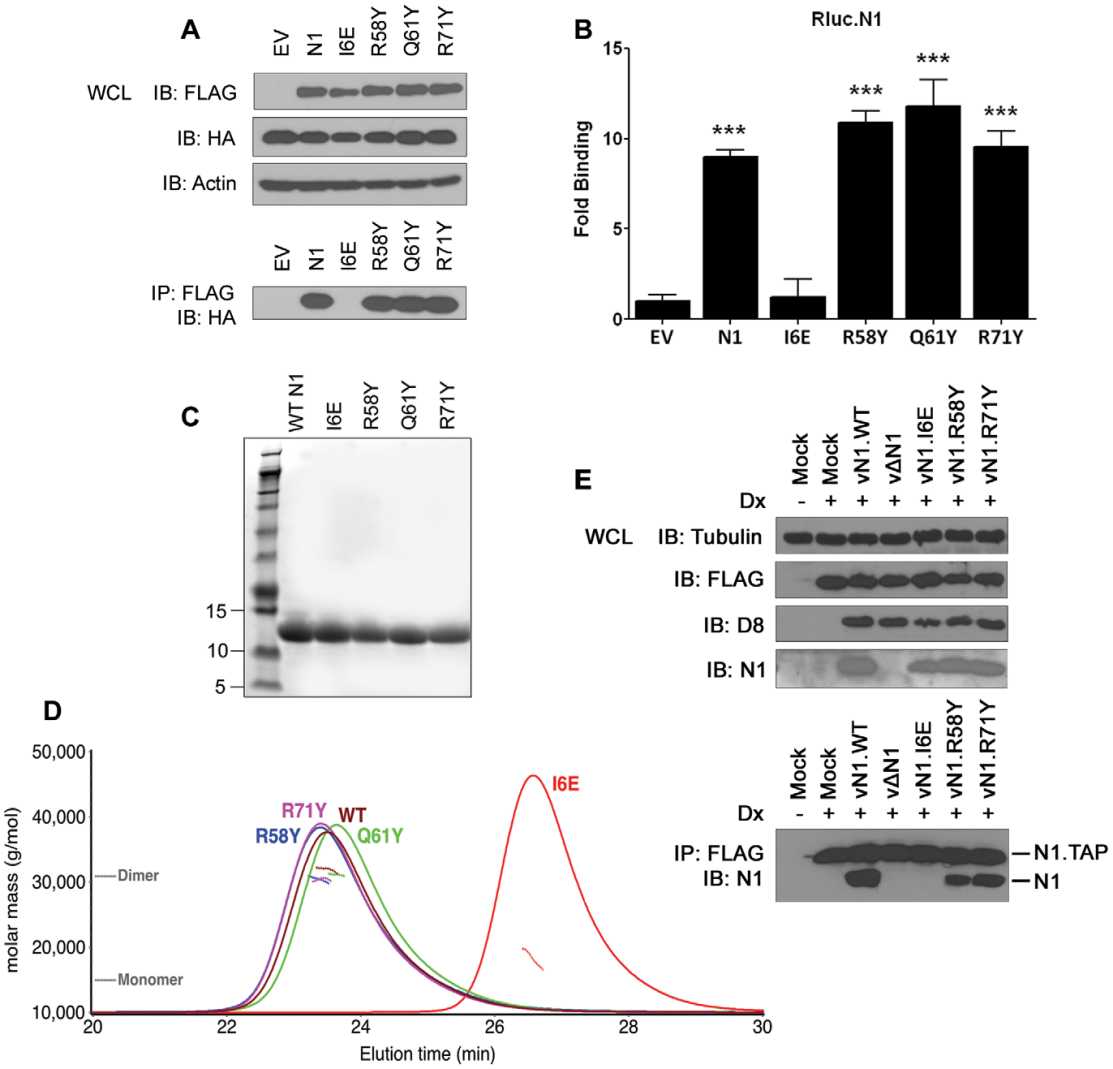
Biochemical and biophysical characterization of mutant N1. (**A**) Ability of FLAG-tagged WT and mutant N1 to immunoprecipitate HA-tagged WT N1 after transfection in HEK 293T cells. (**B**) Ability of FLAG-tagged WT and mutant N1 to immunoprecipitate a renilla luciferase-fused WT N1 (Rluc.N1). Relative fold binding for each plasmid is calculated in triplicates after normalization to empty vector (EV). Data are expressed as means ± SD with statistical analysis (Student's *t*-test; ***P<0.0005). (**C**) Purification of WT and mutant N1 after over-expression in *E. coli*. (**D**) SEC-MALS curves obtained for WT and mutant N1. Weight-averaged molar mass (dotted lines) is shown across the elution profile (A280 nm, solid lines) of wild-type and mutant N1. While wild-type (WT) N1 and the groove-filling mutants elute as a dimer, I6E N1 is predominantly monomeric. (**E**) Ability of mutant N1 proteins expressed from recombinant VACV viruses to dimerise in HEK 293 T-REx cells with TAP-tagged N1 expressed after addition of doxycycline (Dx) for 2 h.

The ability of the N1 mutants to form dimers was investigated next. Plasmids encoding FLAG-tagged wild-type (WT) or mutant N1 were co-transfected into HEK 293T cells with HA-tagged WT N1. FLAG- and HA-tagged mutant N1 proteins were expressed to the same level as WT N1 (Figure 3A). After immunoprecipitation (IP) with an anti-FLAG antibody, the presence of HA-tagged N1 was analysed by immunoblotting with anti-HA antibody. While the surface groove mutants (R58Y, Q61Y, R71Y) and WT N1 interacted efficiently with HA-tagged N1, the I6E mutant did not (Figure 3A). Similar results were obtained by LUMIER after co-transfection of the FLAG-tagged N1 alleles together with WT N1 fused with renilla luciferase (Rluc.N1) (Figure 3B). After measuring the luciferase activity in the cell lysates and the immunoprecipitates using anti-FLAG monoclonal antibody, a ratio for each condition was calculated and compared to mock controls. The I6E mutant was the only protein unable to interact with Rluc.N1. To quantify the extent of self-association we measured the molar mass of WT and mutant N1 directly by subjecting purified proteins (Figure 3C) to size-exclusion chromatography with multi-angle light scattering (SEC-MALS). While WT N1 and the groove-filling mutants (R58Y, Q61Y, R71Y) were all dimeric, the I6E mutant was almost exclusively monomeric (Figure 3D). The ability of the mutant N1 proteins to dimerise during viral infection was also investigated using an inducible cell line expressing TAP-tagged WT N1 and VACV recombinant viruses expressing mutant N1 (see below for virus generation and characterisation). Cells were induced to express N1 by addition of doxycycline and subsequently were infected with the viruses expressing mutant N1 proteins (Figure 3E). After IP with anti-FLAG, immunoprecipitates were immunoblotted to reveal the presence of the immunoprecipitated FLAG-tagged N1 from the cell line and its association with untagged N1 protein from the viruses. WT, mutant R58Y and R71Y N1 proteins expressed during viral infection retained ability to dimerise, whereas mutant I6E N1 protein did not. Taken together, these results show that I6E N1 does not self-associate either *in vitro* or in mammalian cells.

### Functional characterization of mutant N1

**Figure 4:**
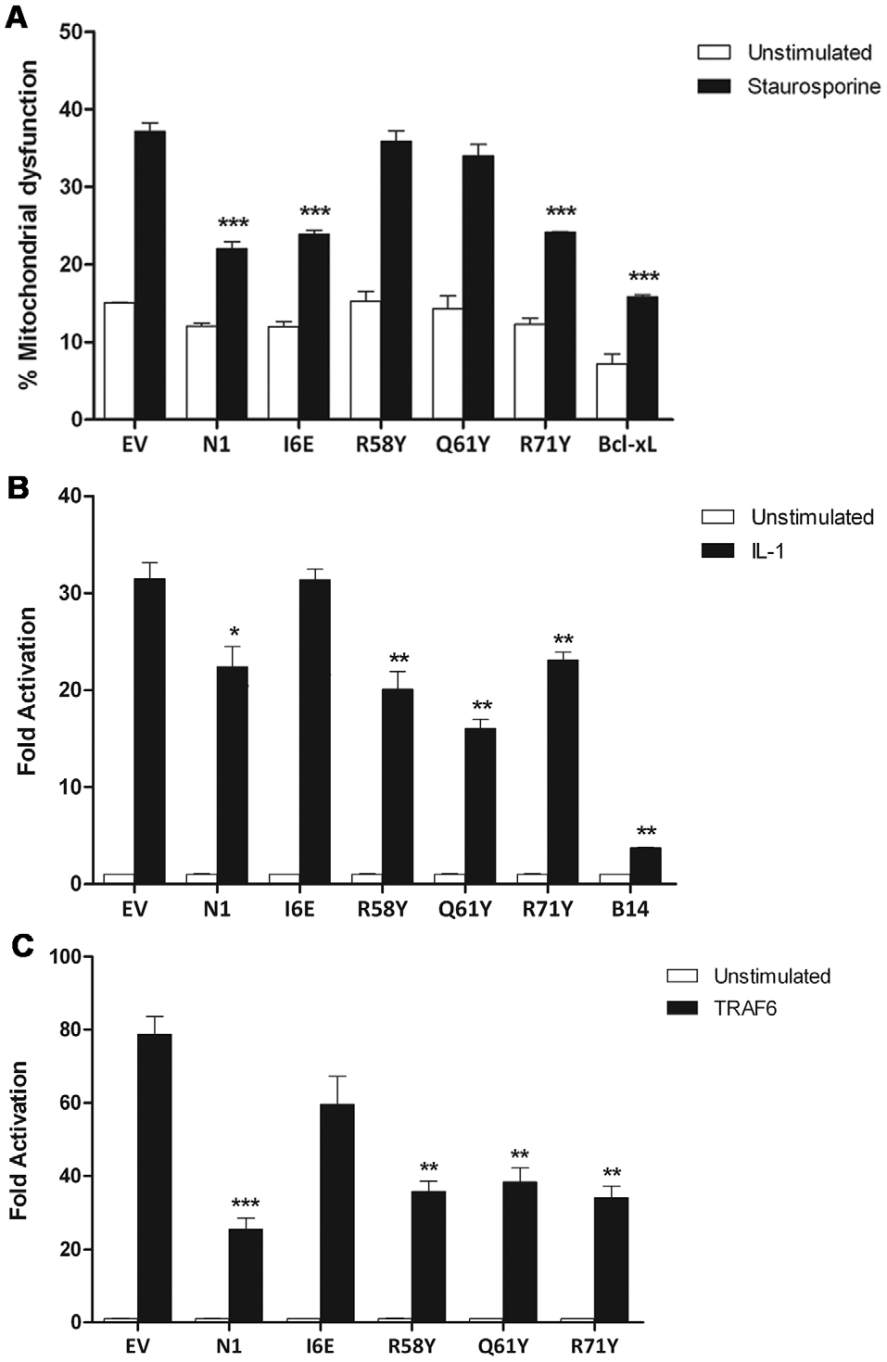
Functional characterization of mutant N1. (**A**) Inhibition of apoptosis by measurement of mitochondrial dysfunction in cells transfected with wild-type and mutant N1, after treatment with staurosporine (black bars) or mock-treated (white bars). (**B**) Inhibition of NF-κB activation in cells co-transfected with reporter plasmids for NF-κB activity and TK-Rluc as internal control, together with 100 ng of the indicated plasmids or pcDNA4/TO (EV). Cells were treated with IL-1β, lysed and the relative fold activation of NF-κB activity was determined. (**C**) Alternatively, 10 ng of FLAG-TRAF6 plasmid was included in the transfection mix to induce activation of the NF-κB reporter. Data is expressed as means ± SD with statistical analysis (Student's *t*-test; *P<0.05; **P<0.005; ***P<0.0005).

The anti-apoptotic ability of the N1 mutants was analysed next. FLAG-tagged plasmids expressing WT and mutant N1 were co-transfected in HeLa cells together with a CD20 surface marker. Mutant and WT proteins showed a comparable expression in HeLa cells (Figure S1), as previously observed for HEK 293T cells. Cells were treated with STS and the permeabilization of the mitochondrial membrane was measured in CD20 positive cells. Introduction of the groove-filling mutations R58Y and Q61Y abolished the ability of N1 to prevent apoptosis, while mutants R71Y and I6E inhibited STS-induced apoptosis as strongly as WT N1 (Figure 4A). As the mutated side chains were the only significant structural difference between wild-type N1 and the mutants that failed to inhibit apoptosis, these data demonstrate that the surface groove determines the ability of N1 to inhibit apoptosis and that N1 dimerisation is not required for inhibition of apoptosis.

We next sought to determine the ability of the N1 mutants to interfere with NF-κB signalling. HEK 293T cells were transfected with an NF-κB-luciferase responsive reporter alongside the different FLAG-tagged N1 mutants and these cells were stimulated subsequently with IL-1β. Expression of WT N1 decreased NF-κB activation significantly, as did expression of the groove mutants (Figure 4B). However, expression of the I6E mutant did not inhibit NF-κB-induced gene expression significantly. NF-κB activation was also induced by over-expression of TRAF6 and under these conditions the different FLAG-tagged N1 mutants gave similar results (Figure 4C). These data show that I6E N1 is impaired for inhibition of NF-κB activation, and demonstrates that mutagenesis in the BH3-binding cleft does not interfere with the ability of N1 to inhibit inflammatory signalling. These data might indicate that either dimer formation is needed for the ability of N1 to inhibit NF-κB activation, or that the dimer interface is needed for binding of unknown signalling molecules that activate NF-κB and this, as well as dimer formation, is ablated by the I6E mutation.

### Immunoprecipitation of pro-apoptotic Bcl-2 proteins with mutant N1 proteins

**Figure 5:**
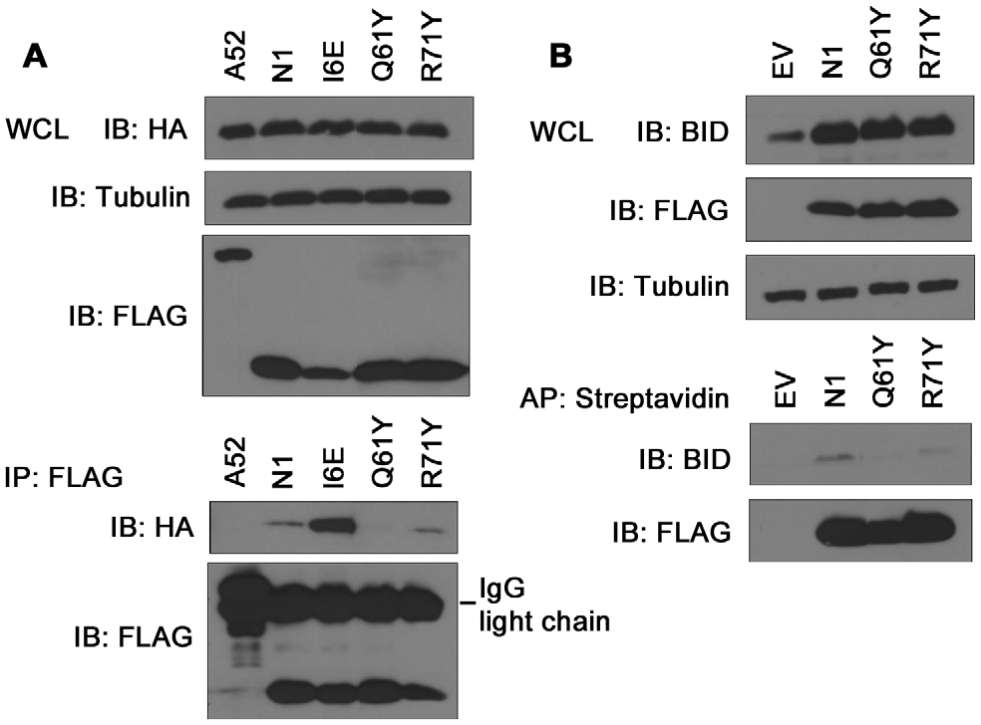
Immunoprecipitation of N1 mutants with cellular pro-apoptotic Bcl-2 proteins. (**A**) Ability of TAP-tagged WT and mutant N1 to immunoprecipitate HA-tagged Bad after transfection in HEK 293T cells. FLAG-tagged N1 constructs were immunoprecipitated with anti-FLAG antibody and presence of HA-tagged Bad was determined by anti-HA immunoblotting. (**B**) Ability of TAP-tagged WT and mutant N1 to immunoprecipitate Bid after transfection in HEK 293T cells. TAP-tagged N1 constructs were affinity-purified (AP) using streptavidin beads and presence of Bid was analysed by anti-Bid immunoblotting.

Previously, N1 was shown to interact with cellular pro-apoptotic Bcl-2 proteins [6], [8]. To determine whether the loss of anti-apoptotic activity of groove-filling N1 mutants R58Y and Q61Y correlated with loss of binding to pro-apoptotic Bcl-2 proteins, immunoprecipitation assays were carried out. HA-tagged Bad was co-expressed in 293T cells together with FLAG-tagged mutant N1 proteins and after IP with anti-FLAG antibody, association with Bad was revealed by anti-HA immunoblotting (Figure 5A). VACV protein A52 was used as negative control because the surface groove of this protein is occluded and A52 is not able to inhibit apoptosis [7]. In this assay, both WT and I6E mutant N1, where the groove is intact, interacted with HA-tagged Bad, whereas groove-filling Q61Y mutant N1, as well as A52, failed to immunoprecipitate with Bad. Interestingly, groove mutant R71Y which retains the ability to prevent STS-mediated apoptosis, associated with Bad. Likewise, when untagged Bid was co-expressed in cells, Q61Y mutant was unable to interact with Bid, whereas R71Y mutant, as well as WT N1, still immunoprecipitated Bid (Figure 5B). Similar results were obtained with the use of VACV recombinant viruses expressing these mutant proteins (data not shown). The interaction of WT and mutant N1 proteins with Bax was also investigated, but under the conditions tested the level of interaction with Bax was too low to enable the binding of the mutant N1 protein to be determined. Collectively, these results indicate that mutations in the N1 groove that inhibited anti-apoptotic activity correlate with an inability to interact with cellular pro-apoptotic Bcl-2 proteins.

### Mitochondrial targeting of N1 abolishes its anti-apoptotic function

**Figure 6:**
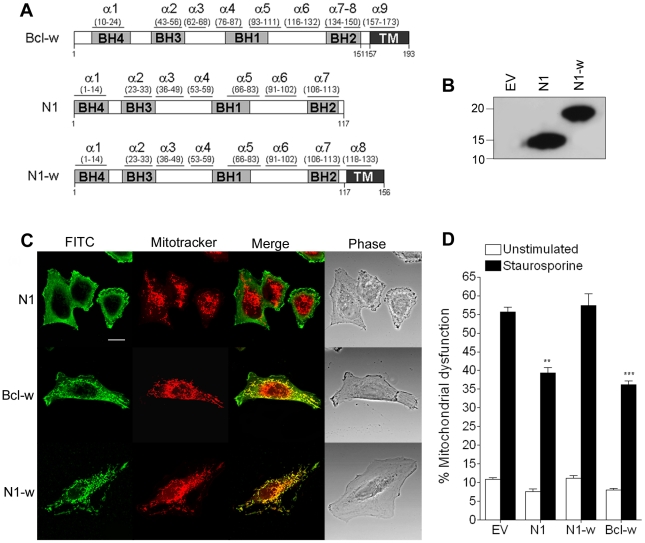
Mitochondrial targeting of N1 abolishes its anti-apoptotic function. (**A**) Addition of the mitochondrial-targeting C-terminus domain from Bcl-w into N1 to generate N1-w. (**B**) Immunoblot showing the expression levels of WT N1 and N1-w in transfected cells. (**C**) Confocal images showing localisation of N1, N1-w and Bcl-w after transfection into HeLa cells incubated with Mitotracker Red and stained with anti-N1 and FITC-conjugated anti-rabbit antibody. (**D**) Mitochondrial dysfunction measurement in cells transfected with N1, N1-w and Bcl-w after treatment with staurosporine (black bars) or mock-treated (white bars). Data are means ± SD with statistical analysis (Student's *t*-test; **P<0.005; ***P<0.0005).

Cellular anti-apoptotic Bcl-2 proteins, like Bcl-x_L_ and Bcl-w, reside in the OMM where they prevent cell death by binding BH3 peptides of pro-apoptotic Bcl-2 family proteins [36]. In contrast VACV protein N1 is cytosolic without localisation to the mitochondrion or other organelles [3]. To address whether the ability of N1 to interfere with pro-apoptotic signalling could be enhanced if it were present on mitochondria, the last 38 residues of Bcl-w, which target Bcl-w to mitochondria, were fused to the C terminus of N1 (Figure 6A). The chimeric protein (N1-w) was expressed at similar levels to WT N1 (Figure 6B) and, like Bcl-w, localised exclusively to mitochondria, whereas WT N1 had a diffuse cytoplasmic localisation (Figure 6C) as seen previously [3]. However, N1-w was unable to protect cells from STS-induced apoptosis, whereas both N1 and Bcl-w could do so (Figure 6D). This suggests that N1 requires a cytosolic localisation to interfere with cell death signalling.

### Abolition of the N1 anti-NF-κB activity, but not the anti-apoptotic activity, attenuates VACV virulence

Having identified N1 mutants displaying exclusively an anti-NF-κB or anti-apoptotic phenotype, we generated recombinant VACV in which these alleles were reinserted into a deletion mutant lacking the *N1L* gene (vΔN1) at the *N1L* gene natural locus [3]. Viruses expressing N1 I6E (vN1.I6E), R58Y (vN1.R58Y), or R71Y (vN1.R71Y) were constructed via transient dominant selection (see Materials and Methods). The genomes of these viruses were investigated by PCR using primers either side of the *N1L* gene locus and the DNA sequence of these PCR products was determined, confirming the presence of individual mutations and no other changes. The ability of these viruses to replicate in cell culture was characterised and there were no growth differences compared to wild-type virus (data not shown), as would be expected since no differences were observed between carefully matched recombinant viruses with or without the *N1L* gene [3]. The expression of the N1 protein by these viruses was investigated by immunoblotting of infected cell extracts and this showed that the proteins were expressed at similar levels to wild-type (data not shown), as had been observed following transfection (Figure 3A and Figure S1).

**Figure 7:**
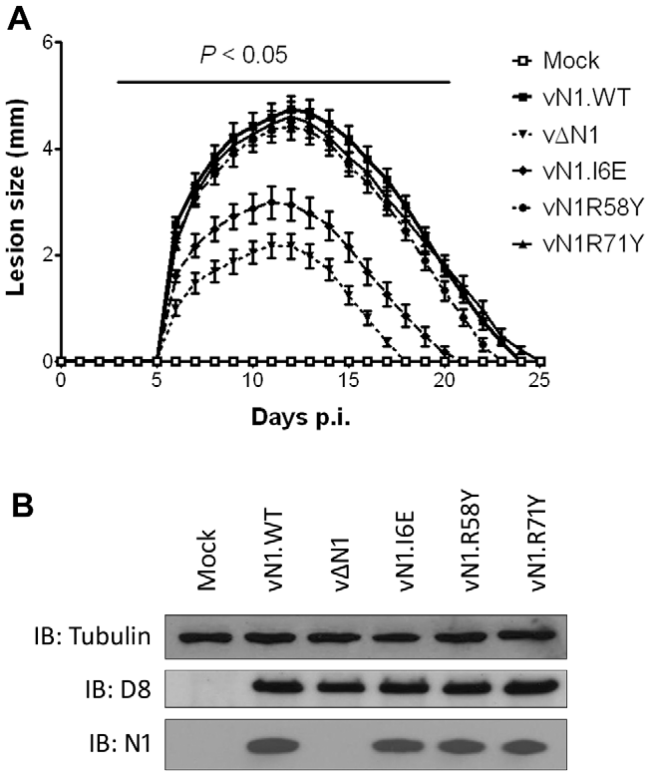
Intradermal inoculation with vN1.I6E and vΔN1, but not vN1.R58Y and vN1.R71Y, attenuates VACV infection. (**A**) Size of the lesions observed daily after infection of mice (groups of 5) by intradermal injection of 10^4^ p.f.u. of the indicated virus in each of the ear pinnae. Data shown are the mean ± standard error of the mean (s.e.m.) of lesion sizes for each group of animals. The horizontal bar indicates the days on which the lesion size caused by vΔN1 and vN1.I6E was statistically different from vN1.WT (P<0.05, Student's t-test). (**B**) Expression levels of WT and mutant N1 compared to VACV control protein D8 in ear pinnae tissues after 48 h p.i.

The mutant N1 viruses were then used to investigate the contribution of N1-mediated anti-apoptotic activity or inhibition of NF-κB activation to virulence. Groups of mice were infected with the N1 mutant viruses by either the intradermal or intranasal route and compared with wild-type virus and vΔN1. In the intradermal model, animals were inoculated with 10^4^ plaque-forming units (p.f.u.) in each ear and the size of the local lesions formed was recorded daily (Figure 7A). Inoculation with vΔN1 generated significantly smaller lesion sizes than the control wild-type virus (vN1.WT) from days 6 to 25 post infection (p.i.). However, infection with vN1.R58Y or vN1.R71Y induced lesions that were indistinguishable from vN1.WT. In contrast, lesions induced by vN1.I6E were smaller than those obtained for vN1.WT (*p*<0.05 on days 6 to 25 p.i.) and were similar to those induced by vΔN1. To check the level of infection with these viruses were equivalent, and to investigate if the mutant N1 proteins were stable *in vivo*, the amount of N1 and a late structural protein, D8, present within infected tissue 48 h p.i. was investigated by immunoblotting. This showed that the levels of N1 and D8 expression were equivalent between the different viruses except for vΔN1, which produced no N1 as expected (Figure 7B). Therefore, the different phenotypes observed *in vivo* between the N1 mutants were not due to different levels of N1 protein.

**Figure 8:**
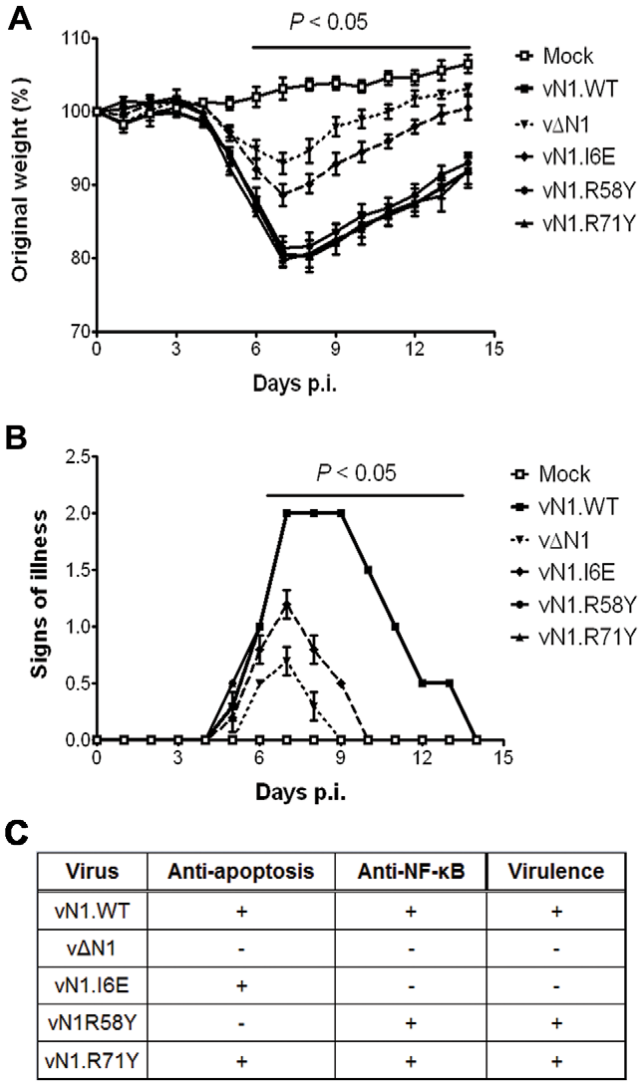
Intranasal inoculation with vN1.I6E and vΔN1, but not vN1.R58Y and vN1.R71Y, attenuates VACV infection. (**A**) Weight of mice (groups of 5) infected intranasally with 5×10^3^ p.f.u. of the indicated virus. The mean weight of each group on each day is expressed as ratio of the mean weight of the same group on day zero. Data shown are the mean ± s.e.m. of relative weight for each group of animals. (**B**) Signs of illness recorded for every group according to an arbitrary scale of 1 to 4 as described previously [62]. The horizontal bar indicates the days on which the weight loss or the signs of illness caused by vΔN1 and vN1.I6E was statistically different from vN1.WT (P<0.05, Student's t-test). (**C**) Summary table of the biochemical functions and *in vivo* virulence of the N1 alleles introduced into VACV strain Western Reserve (WR) compared to wild-type (vN1.WT) and deletion mutant (vΔN1, [3]).

In the intranasal infection model, inoculation with vΔN1 induced less weight loss and reduced signs of illness compared with vN1.WT, and these differences were significant from days 6 to 15 p.i. (Figure 8A and B). Inoculation with vN1.R58Y and vN1.R71Y caused a similar outcome as vN1.WT, indicating these mutations did not result in virus attenuation. Inoculation with vN1.I6E, however, resulted in lower weight loss and reduced signs of illness, similar to the loss observed in animals infected with vΔN1.

Collectively, vN1.I6E, but not vN1.R58Y or vN1.R71Y, was attenuated in both murine models of infection. Considering that R58Y N1 was no longer anti-apoptotic and I6E N1 no longer displayed anti-NF-κB activity, our results indicate that suppression of the N1 anti-apoptotic activity does not significantly attenuate VACV virulence whilst suppression of NF-κB activation does.

## Discussion

VACV protein N1 is an intracellular virulence factor that is expressed early during infection and modulates innate immunity by at least two strategies: preventing apoptosis and inhibiting NF-κB activation [7], [8], [13]. In this study, structurally-informed mutagenesis and functional analysis were used to identify distinct regions of the N1 protein that are required for these different activities, and, using recombinant viruses engineered to express N1 proteins that inhibit either apoptosis or NF-κB activation, the relative contribution of these activities to virus virulence was determined.

N1 is a Bcl-2-like protein that contains a surface groove [6], [8] similar to those in cellular anti-apoptotic Bcl-2 proteins that bind the BH3 motif of pro-apoptotic Bcl-2 members [36], [37]. Like other cellular anti-apoptotic Bcl-2 proteins, N1 can also bind some pro-apoptotic Bcl-2 proteins and BH3 peptides [6], [8]. To determine if N1 mediated its anti-apoptotic activity via the surface groove, tyrosine residues were introduced into the groove by mutagenesis. Protein crystallography showed that 2 of these mutations (R58Y and Q61Y) placed the bulky aromatic side chain of the tyrosine residue within the groove in a position likely to restrict access by BH3 peptides, but in other respects these mutant proteins were unchanged from wild-type (Figure 2 and Figure S2). In contrast, a R71Y mutation placed the tyrosine flat on the base of the groove. Notably, the N1 mutants R58Y and Q61Y were unable to block STS-induced apoptosis, whereas R71Y, wild-type N1 and also an I6E mutant, which could no longer form dimers, did so (Figure 4A). Consistent with this, WT N1 and mutant I6E and R71Y all retain an open surface groove (Figure 2), bind Bid and Bad (Figure 5), and inhibit apoptosis (Figure 4). In contrast, mutant Q61Y which has an occluded surface groove (Figure 2) no longer binds Bid or Bad (Figure 5) and no longer inhibits apoptosis (Figure 4). Investigation of N1 mutant R58Y binding to Bid and Bad was unsuccessful because this mutant bound strongly to all proteins examined, including both pro-apoptotic and anti-apoptotic Bcl-2 proteins and tubulin, for unknown reasons (data not shown). In conclusion, the surface groove is critical for the anti-apoptotic activity of N1 whereas dimer formation is not.

Programmed cell death requires the activation of Bax and Bak (the pro-apoptotic effector proteins), which is proposed to occur by one of two mechanisms: the ‘direct activation’ model, in which BH3-only proteins directly interact with Bax and Bak [40], and the ‘indirect activation’ model, where the BH3-only proteins bind to the anti-apoptotic Bcl-2 proteins inducing the release of Bax and Bak [41]. Bak is a mitochondrial integral membrane protein and binds Bcl-x_L_ [42], but Bax is a soluble protein that requires insertion to the OMM for activation and is normally held in the cytoplasm by anti-apoptotic Bcl-2 members [43], [44]. Data presented here suggest that N1 exerts its anti-apoptotic function in the cell cytoplasm, but not at the OMM (Figure 6), targeting pro-apoptotic cellular BH3-only Bcl-2 proteins by virtue of its BH3-binding groove (Figure 5). This strategy differs from those reported for other viral anti-apoptotic proteins, such as VACV F1 or myxoma virus M11, that directly bind to mitochondrial-bound Bak [28], [31], [32], [33], [45].

An unusual feature that distinguishes N1 from other viral anti-apoptotic proteins is its ability to also inhibit pro-inflammatory signalling, particularly NF-κB activation [7], [8], [13]. In this study, we have identified a mutation in N1 (I6E) that abolishes its ability to form dimers (Figure 3) and its anti-NF-κB activity (Figure 4B and 4C), while retaining its anti-apoptotic potency (Figure 4A) and its ability to bind Bid and Bad (Figure 5). An obvious conclusion would be that dimerisation of N1 is required for its ability to block NF-κB activation. However, in other viral and cellular Bcl-2 family proteins the surface equivalent to the N1 dimer interface, formed by helices 1 and 6, has been implicated in binding partner proteins [11], [39], [46]. In the case of B14, mutation of this surface inhibited the ability of B14 to bind IKKβ and block NF-κB activation [11]. It is possible that the ‘1–6 face’ of N1 mediates its binding to other proteins, and that dimerisation is driven by a requirement to shield this hydrophobic protein:protein interaction surface from polar solvents. Interestingly, I6E N1 was not completely impaired for NF-κB inhibition and retained minimal inhibitory activity when compared to control samples (Figure 4C). VACV containing the I6E mutation on N1 (vN1.I6E) also was slightly less attenuated than vΔN1 (Figure 7 and 8). It is therefore possible that while the I6E mutation significantly disrupted dimerisation, it did not completely abolish N1 interaction with a binding partner that mediates inhibition of NF-κB activation. Confirmation of this will require identification of N1 binding partners and understanding of the molecular mechanism by which N1 inhibits the NF-κB pathway.

Deletion of the VACV *N1L* gene attenuates VACV virulence [3], [4], [5], [47]. To understand the mechanism underlying this attenuation, we have analysed the virulence of VACVs expressing an N1 protein with significantly reduced anti-apoptotic (vN1.R58Y) properties, or anti-inflammatory properties (vN1.I6E), or a protein retaining both activities (vN1.R71Y) and compared these to WT VACV strain Western Reserve (vN1.WT) and a deletion mutant lacking the *N1L* gene (vΔN1). We only observed an attenuated infection upon inoculation with vN1.I6E, which resembled that of vΔN1 (Figure 7 and 8). Mice infected with vN1.I6E lost less weight, and presented reduced signs of illness in the intranasal model and developed smaller lesions in the intradermal model of infection. In contrast, vN1.R58Y and vN1.R71Y were as virulent as vN1.WT, possibly because apoptosis is inhibited by other VACV anti-apoptotic proteins [31]–[33]. These results demonstrate that the virulence of VACV WR is not increased by the anti-apoptotic activity of the N1 protein. Virulence is instead enhanced by the presence of N1 dimers and/or an intact N1 dimerisation interface facilitating N1-mediated inhibition of NF-κB dependent inflammatory signalling and potentially other (yet unknown) cellular signalling pathways.

It is interesting that the inhibition of NF-κB activation by N1 is so important for virulence while the virus expresses several other proteins that inhibit NF-κB activation. There may be several reasons for this but one important factor is likely to be the position in the signalling pathway at which each virus inhibitor acts. For instance, an inhibitor of TLR- and IL-1-induced NF-κB activation could contribute to virulence differently to an inhibitor of TNF-induced NF-κB activation, and be different again to an inhibitor, like B14, that blocks all these pathways at the IKK complex [9]. Further, there is cross talk between signalling pathways and an NF-κB inhibitor acting at the IKK complex would not inhibit mitogen-activated protein kinase (MAPK) activation following TNF stimulation, whereas an inhibitor acting at the TNF receptor complex might do so.

The severe attenuation of VACV lacking N1 has been linked to a stronger natural killer cell response and a modulation of CD69^+^ cells [4], but also to a more robust intrapulmonary CD8^+^ T cell response [47]. More recently, Gratz et al. (2011) could only observe disease caused by infection of an ectromelia virus (the causative agent of mousepox) lacking the *N1L* gene after depletion of both CD4^+^ and CD8^+^ T cells, thus linking N1 activity to modulation of the T cell function [48]. While it seems that the attenuation of vΔN1 can be attributed to T cell function, an immune response generated very early during the infection precedes and determines this adaptive response. We show here that pro-inflammatory signalling rather than the apoptotic signalling pathway is crucial for the orchestration of the adaptive immune response that leads to the clearance of vΔN1. Presumably, an NF-κB-dependent sensing of VACV infection induces a robust production of pro-inflammatory cytokines and chemokines that eventually modulate the adaptive response. Therefore, an NF-κB-dependent immune activation is important for the control of VACV infection.

In summary, we identified mutations at the surface groove and the dimer interface of N1 that interrupt either its ability to inhibit host-cell apoptosis or to inhibit the NF-κB pathway. This proves that these two activities are distinct and mediated by discrete regions of the protein surface. Only mutations that disrupt inhibition of NF-κB activation lead to significant attenuation of VACV following intranasal and intradermal infection in mice. These results show that the ability of N1 to prevent apoptosis does not contribute to VACV WR virulence in the models studied and highlights the crucial role played by NF-κB in development of immune responses against viral infection.

## Material and Methods

### Ethics statement

This work was carried out in accordance with regulations of The Animals (Scientific Procedures) Act 1986. All procedures were approved by the United Kingdom Home Office and carried out under the Home Office project licence PPL 70/7116.

### Expression plasmids and antibodies

pET24a-based bacterial expression plasmids containing C-terminal His-tagged N1 and N1(C40S) (pET24a-N1 and pET24a-N1-C40S respectively) were described previously [3], [8]. A mammalian expression plasmid of N1 incorporating a C-terminal FLAG tag was generated by PCR amplification of cDNA from pET24a-N1 using Platinum Taq HiFi DNA polymerase (Invitrogen) with forward primer 5′-CGCGAATTCGCCACCATGAGGACTCTACTTATTAGATATATTCTTG-3′ (EcoRI site underlined) and reverse primer 5′- GCCTCTAGATTACTTATCGTCGTCATCCTTGTAATCTTTTTCACCATA-TAGATCAATCATTAGATC -3′ (XbaI site underlined) containing the FLAG sequence. The PCR product was cloned into the tetracycline-inducible expression vector pcDNA4/TO (Invitrogen) via EcoRI and XbaI (Roche) restriction sites using T4 DNA ligase (Novagen) to make pcDNA4/TO-N1-FLAG. Also, the N1 cDNA was cloned into pcDNA4/TO incorporating a C-terminal HA tag. Alternatively, the sequence of N1 was codon-optimised (coN1) for expression in human cells (GENEART) and subcloned into pcDNA4/TO as a fusion to C-terminal tandem-affinity purification (TAP) tag containing 2 copies of the streptavidin-binding sequence and 1 copy of the FLAG epitope [49].

The renilla luciferase (Rluc)-fused N1 expression vector was generated by PCR amplification of N1 cDNA with forward primer 5′-GGCTCATGAGGACTCTACTTATTAGA-3′ (BspHI site underlined) and reverse primer 5′-GCAGCGGCCGCTTATTTTTCACCATATAGATC-3′ (NotI site underlined). The PCR product was cloned downstream the *Rluc* gene present in the M5P vector (a gift from Dr. Felix Randow, MRC Laboratory of Molecular Biology, Cambridge, United Kingdom, [50]) that had been digested with PciI and NotI to generate an N-terminal Rluc.N1 fusion.

The FLAG-tagged B14 expression plasmid was described previously [51]. NF-κB luciferase reporter, pTK-renilla luciferase, and FLAG-tagged TRAF6 were gifts from Dr. Andrew Bowie (Trinity College, Dublin, Ireland). Bcl-x_L_ was provided by Professor Xin Lu (Ludwig Institute for Cancer Research, Oxford, United Kingdom). CD20 plasmid was provided by Dr. Nick Dyson (Charleston, USA). Anti-N1 [3], anti-D8 [52], anti-FLAG (Sigma), anti-HA (Sigma), anti-Bid (Cell Signaling), anti-tubulin (Millipore) and anti-actin (Sigma) antibodies were used to determine protein expression levels.

### Mutagenesis of N1

To construct an N1 mutant with a mitochondrial-targeting C-terminal hydrophobic tail, a structure-based sequence alignment of N1 and human Bcl-w was used to determine the additional tail residues (G155-K193) present in Bcl-w but absent in N1. To generate an N1/Bcl-w tail fusion product (N1-w), N1 was amplified by PCR from pET24a-N1 Platinum Taq HiFi DNA polymerase (Invitrogen) with the forward primer described above for generation of pcDNA4/TO-N1-FLAG and reverse primer 5′-**CTCCTCCAGGGC** TTTTTCACCATATAGATCAATCATTAG-3′ (N1 sequence is underlined; Bcl-w sequence in bold). For the Bcl-w tail, human mRNA was extracted from HeLa cells using an RNeasy purification kit (Qiagen). Bcl-w cDNA was generated using Superscript III reverse transcriptase (Invitrogen) according to the manufacturer's instructions and gene-specific reverse primer 5′-GCCTCTAGA ***TCA*** **CTTGCTAGCAAAAAAGGCCCCTAC**-3′ (XbaI site is underlined; Bcl-w sequence is in bold; stop codon in bold italic). The Bcl-w tail was amplified from the Bcl-w cDNA using forward primer 5′-TATGGTGAAAAA **GCCCTGGAGGAGGCGCGGCGTC**-3′ (N1 sequence is underlined, Bcl-w sequence is in bold) and the reverse primer used above for generation of Bcl-w cDNA. N1-w was generated by PCR using equimolar amounts of N1 and Bcl-w tail PCR products as templates, purified using a QIAquick PCR purification kit (Qiagen). The PCR was carried out using the forward primer used to construct pcDNA4/TO-N1-FLAG and the reverse primer used for generation of the Bcl-w tail PCR product. The resulting N1-w PCR product was cloned into pcDNA4/TO (Invitrogen) via EcoRI and XbaI (Roche) restriction sites using T4 DNA ligase (Novagen) to make pcDNA4/TO-N1-w.

The N1 structure (PDB ID 2UXE) and a model of the structure overlayed onto the M11:Bak BH3 peptide complex (PDB ID 2JBY) were used to design mutations to disrupt the N1 dimer and fill the N1 BH3 surface groove. All mutations were introduced into pET24a-N1(C40S), pcDNA4/TO-N1 and pcDNA4/TO-coN1(C40S) vectors using the QuikChange site-directed mutagenesis kit (Stratagene) and verified by DNA sequencing.

### Purification, biophysical characterisation, crystallisation, diffraction data collection and structure refinement

Wild-type and mutant N1 were over-expressed in *E. coli* and purified as described previously [8]. The identities of purified wild-type and mutant N1 were confirmed by mass spectroscopy (not shown). Purified wild-type and mutant N1 were concentrated to 2.0–2.4 mg mL^−1^ and injected (100 µL) onto an analytical S75 Superdex 10/300 gel filtration column equilibrated in 50 mM Tris, 150 mM NaCl, pH 8.5. Static light-scattering (DAWN HELEOS II, Wyatt Technology), differential refractive index (Optilab rEX, Wyatt Technology) and UV absorbance (280 nm; Agilent 1200 UV, Agilent Techologies) of the eluate were recorded inline and data were analysed using the ASTRA software package (Wyatt Technology).

Purified mutant N1 was further concentrated to 15.4–19.7 mg mL^−1^ in 50 mM Tris, 150 mM NaCl, pH 8.5 and crystallised in sitting drops containing 100–200 nL of protein and 100 nL of reservoir solution (R58Y: 2% w/v polyethylene glycol 3350, 15% w/v Tacsimate pH 7.0, 100 mM HEPES pH 7.0; Q61Y and R71Y: 19–21% PEG 1500, 76–85 mM MIB buffer system (Molecular Dimensions) pH 7.0) equilibrated against 95 µL reservoirs at 20.5°C. Crystals were cryoprotected by brief immersion in reservoir solution supplemented with 20–25% v/v glycerol before flash-cryocooling in a cold (100 K) N2 gas stream. Diffraction data were recorded from cryocooled (100 K) crystals at Diamond beam line I03 (R71Y) and ESRF beam lines ID23-2 (R58Y) or ID14-2 (Q61Y). Diffraction data were processed using HKL2000.

Structures of N1 mutants were solved by isomorphous difference fourier synthesis, using the high resolution structure of N1 (PDB ID 2I39) [8] as a starting model. Structures were built using COOT [53] and refined in BUSTER-TNT (Global Phasing) using TLS and Local Structure Similarity Restraints [54]. The refinement was informed by the MolProbity web server [55] and the validation tools present in COOT. Structural superpositions were performed using SSM [56] and molecular graphics were prepared using PyMOL (DeLano Scientific).

### Cells and viruses

BS-C-1, HeLa, HEK 293T and HEK 293 T-REx cells (Invitrogen) were grown at 37°C in a 5% CO_2_ atmosphere in Dulbecco's modified Eagle's medium (DMEM), or minimum essential medium (MEM) for HeLa cells, supplemented with 10% fetal bovine serum (FBS; Invitrogen), 4 mM L-glutamine (Invitrogen), 100 U mL^−1^ penicillin (Invitrogen) and 100 mg mL^−1^ streptomycin (Invitrogen). HeLa cells and HEK 293 T-REx cells were also supplemented with MEM non-essential amino acid solution (Sigma) and blasticidin (Invitrogen), respectively. A stable cell line expressing TAP-tagged N1 in an inducible manner was generated by transfection of a TAP-tagged N1 construct (see section of expression plasmids) into HEK 293 T-REx cells. Clones were selected in the presence of zeocin (Invitrogen) for over 2 weeks and expression of N1 was shown to be dependent upon addition of doxycycline.

VACV recombinants vN1.WT and vΔN1 derived from VACV strain Western Reserve (WR) were described previously [3]. For the generation of VACV recombinants expressing mutated N1 proteins, the mutations I6E, R58Y and R71Y were introduced by using the QuikChange site-directed mutagenesis kit (Stratagene) into a pUC13 plasmid containing the entire N1 open reading frame and its left and right flanking regions in WR. This plasmid allows transient dominant selection of the desired viruses by expressing *E. coli* guanine xanthine phosphoribosyltransferase (Ecogpt) as selectable marker [57], [58]. All mutations were verified by DNA sequencing. A pUC13-N1 plasmid containing the I6E, the R58Y or the R71Y was then transfected separately into vΔN1-infected cells and mycophenolic acid-resistant viruses were isolated by plaque assay. These viruses were subsequently resolved into deletion virus (vΔN1) or mutant viruses (vN1.I6E, vN1.R58Y, vN1.R71Y) in the presence of 6-thioguanine as described [59]. Viruses were screened by PCR using primers located in the flanking regions, and finally expanded and titrated by plaque assay in BS-C-1 cells. Protein expression was analysed after infection of BS-C-1 cells at 10 p.f.u. per cell for 6 h.

### Immunoflourescence

HeLa cells were seeded on glass coverslips and transfected with expression vectors for wild-type N1, N1-w or Bcl-w using FugeneHD (Roche). Cells were incubated for 30 min with Mitotracker Red (Invitrogen) diluted in MEM containing 2.5% FBS. Cells were immediately fixed with 4% paraformaldehyde (PFA) for 10 min on ice, washed with PBS and fixed again with 8% PFA for 20 min at room temperature. Cells were subsequently quenched with 50 mM ammonium chloride, blocked with 5% horse serum and permeabilised before staining with anti-N1 serum and fluorescein isothiocyanate (FITC)-conjugated anti-rabbit antibody (Invitrogen).

### Apoptosis assay

Apoptosis assays were carried out as described previously [7], [8]. Briefly, HeLa cells were transfected with expression vectors for FLAG-tagged Bcl-x_L_, wild-type or mutant I6E, R58Y, Q61Y, and R71Y N1, or empty vector (EV) pcDNA4/TO together with a plasmid encoding CD20 surface marker using FugeneHD (Roche). Cells were stimulated with 0.5 µM staurosporine for 1 h or left untreated as indicated. The level of apoptosis was assessed by measuring the change in mitochondrial potential (Δψm) using the potentiometric dye JC-1. Cells were collected, washed in phosphate-buffered saline (PBS) and stained with anti-CD20 APC antibody (BD Pharmingen) for 20 min on ice for detection of transfected cells. Then cells were stained with 2 µM JC-1 dye (Invitrogen) for 30 min at 37°C, washed in PBS, re-suspended in FACS buffer (PBS with 2% (v/v) FBS) and analysed by flow cytometry (FACScan; Becton Dickinson). Three independent experiments were performed in triplicates. Data are expressed as means ± standard deviation (SD) with statistical analysis (Student's *t*-test; **P<0.005; ***P<0.0005).

### Reporter gene assays

HEK 293T cells were seeded in 96-well plates and transfected with 100 ng of the indicated expression vectors or pcDNA4/TO (EV), together with 70 ng of an NF-κB-firefly luciferase reporter and 10 ng of pTK-renilla luciferase as internal control, using Fugene6 (Roche). After 24 h, the NF-κB-dependent gene expression was induced by addition of 20 ng mL^−1^ of IL-1β (Peprotech) diluted in DMEM supplemented with 2% FCS. Alternatively, 10 ng of FLAG-tagged TRAF6 plasmid was added into the transfection mix. Cells were then washed once with ice-cold PBS and harvested in passive lysis buffer (Promega). A ratio of firefly:renilla luciferase activity was calculated for every condition and normalized to that of the corresponding unstimulated control to determine the relative fold stimulation of NF-κB activity. At least two experiments were performed in triplicate. Data are expressed as means ± SD with statistical analysis (Student's *t*-test; *P<0.05; **P<0.005; ***P<0.0005).

### Immunoprecipitation and LUMIER

For immunoprecipitation, HEK 293T cells were transfected with a pcDNA4/TO expressing HA-tagged N1 and either TAP-tagged WT or mutant I6E, R58Y, Q61Y, R71Y N1, or empty vector (pcDNA4/TO) using the calcium chloride precipitation method [60]. After 24 h, cells were washed once with ice-cold PBS and lysed with IP buffer (10% glycerol, 150 mM NaCl, 20 mM Tris-HCl [pH 7.4], 0.1% Triton-X100, and protease inhibitors [Roche]). Post-nuclear supernatants were incubated with FLAG agarose (Sigma) for 2 h at 4°C. After 3 washes with ice-cold Tris buffered saline (25 mM Tris-HCl, pH 7.4, 150 mM NaCl, 2 mM KCl, TBS), the beads were boiled in the presence of sample buffer and analysed by immunoblotting. For immunoprecipitation of pro-apoptotic Bcl-2 proteins, the same conditions were used after lysis of cells with an NP-40-based lysis buffer as described in Cooray et al. [8]. Proteins were fractionated and transferred to nitrocellulose membranes (Amersham), blocked for 1 h at room temperature with 5% skimmed milk (Sigma) and incubated with primary antibody overnight at 4°C. The next day, membranes were washed 3 times with PBS containing 0.1% Tween (PBST), incubated with horseradish peroxidase (HRP)-conjugated secondary antibody, washed again with PBST, and finally incubated with chemiluminesence reagent (Amersham) to detect immunoreactive bands.

For LUMIER [61], cells were transfected with the same plasmids, but the HA-tagged N1 was replaced by Rluc.N1. After washing the beads with TBS, beads were incubated with FLAG peptide (Sigma) diluted at 150 µg mL^−1^ in Passive Lysis Buffer (Promega) for 1 h at 4°C. The luciferase activity was measured both in the eluates and the whole cell lysates, and these ratios compared to the ratio of a control reaction were plotted. Data shown are from one out of two independent experiments performed in triplicate. Data are expressed as means ± SD with statistical analysis (Student's *t*-test; ***P<0.0005).

### *In vivo* experiments

The virulence of the recombinant VACVs was analysed in two mouse infection models. In the intranasal model, groups of 5 BALB/C mice 6–8 weeks old were inoculated with 5×10^3^ p.f.u. of the different recombinant virus in 20 µL PBS. Mice were weighed daily and signs of illness were recorded as described previously [62]. In the intradermal model, groups of 5 C57BL/6 mice were inoculated with 10^4^ p.f.u. in 10 µL PBS in both left and right ear pinnae [63]. The size of the lesions in infected ears was monitored daily with the aid of an electronic digital caliper. To determine protein expression levels in tissues after infection, infected ear tissue was collected 48 h p.i., disrupted in 10% (w/v) PBS and centrifuged 500 x *g* for 5 min. Cells were lysed in 0.1 mL of IP buffer and post-nuclear supernatants were treated with benzonase (125 U). Proteins were finally fractionated and analysed by immunoblotting.

## Supporting Information

Figure S1 **Immunoblotting of WT and mutant N1 proteins expressed in HeLa cells.**
(TIF)

Figure S2 **Refined 2Fo-Fc electron density structures of the ‘groove-filling’ mutant N1.**
(TIF)

Table S1 **Data collection and refinement statistics of R58Y, Q61Y and R71Y N1.**
(DOC)
