# Supplementary figures and images for: Inhibition of Apoptosis and NF-κB Activation by Vaccinia Protein N1 Occur via Distinct Binding Surfaces and Make Different Contributions to Virulence
Source: PLoS Pathog. 2011 Dec 15;7(12):e1002430. doi: 10.1371/journal.ppat.1002430 (PMC3240604; doi:10.1371/journal.ppat.1002430)

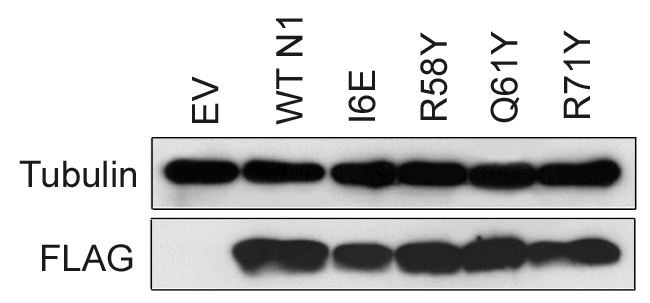

Supplement: Figure S1 — Immunoblotting of WT and mutant N1 proteins expressed in HeLa cells. (TIF) [file ppat.1002430.s001.tif]

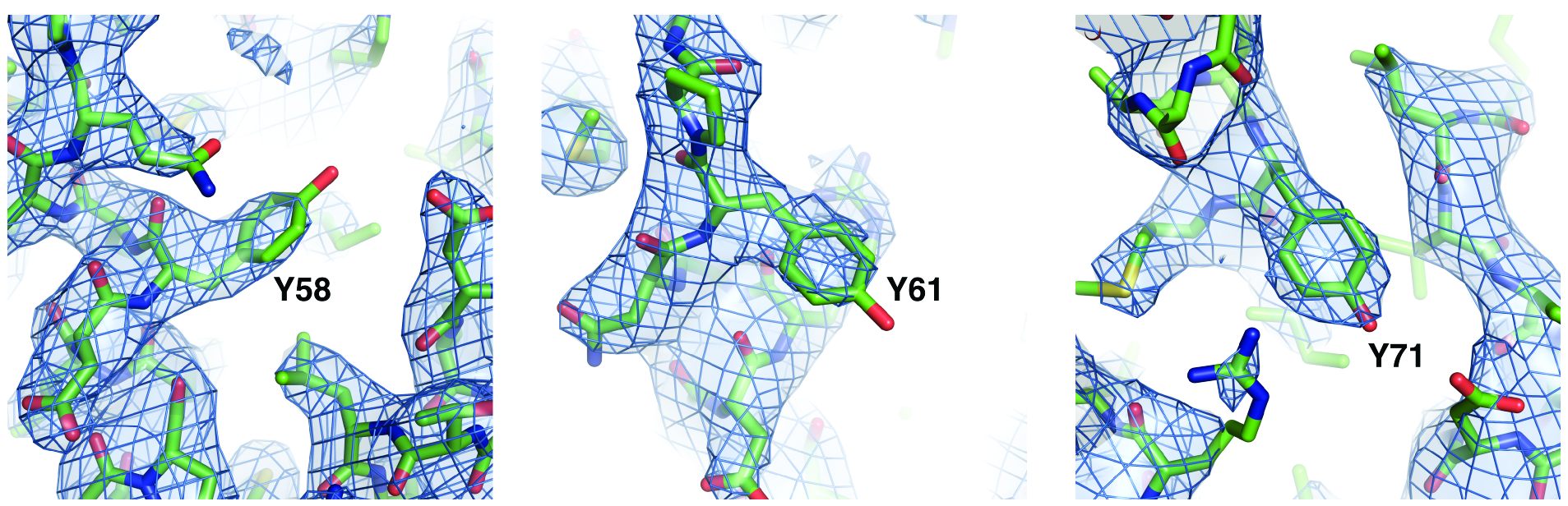

Supplement: Figure S2 — Refined 2Fo-Fc electron density structures of the ‘groove-filling’ mutant N1. (TIF) [file ppat.1002430.s002.tif]
