# Supplementary material for: Inhibition of Apoptosis and NF-κB Activation by Vaccinia Protein N1 Occur via Distinct Binding Surfaces and Make Different Contributions to Virulence
Source: PLoS Pathog. 2011 Dec 15;7(12):e1002430. doi: 10.1371/journal.ppat.1002430 (PMC3240604; doi:10.1371/journal.ppat.1002430)
Supplement: Table S1 — Data collection and refinement statistics of R58Y, Q61Y and R71Y N1. (DOC) [file ppat.1002430.s003.doc]

**Table S1.  Data collection and refinement statistics^a^**

|  | **R58Y N1** | **Q61Y N1** | **R71Y N1** |
| --- | --- | --- | --- |
| **Data collection** |  |  |  |
| Beamline | ESRF ID23-2 | ESRF ID14-2 | Diamond I03 |
| Wavelength (Å) | 0.8726 | 0.9330 | 0.9900 |
| Resolution limits (Å) | 50.0–3.0 (3.05–3.00) | 50.0–3.1 (3.15–3.10) | 50.0–3.1 (3.15–3.10) |
| Space group | *P*2_1_ | *P*2_1_ | *P*2_1_ |
| Unit cell dimensions (Å) | *a*=70.6, *b*=110.2, *c*=72.9, *β*=111.3 | *a*=69.7, *b*=109.8, *c*=70.3, *β*=110.9 | *a*=69.9, =108.9, *c*=71.6, =110.8 |
| Unique reflections | 20,904 (1,057) | 18,168 (898) | 18,280 (900) |
| Redundancy | 7.7 (7.7) | 3.8 (3.8) | 3.8 (3.8) |
| Completeness (%) | 100.0 (100.0) | 100.0 (100.0) | 99.8 (99.6) |
| *I*/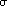(*I*) | 20.0 (2.7) | 13.3 (1.6) | 11.4 (1.7) |
| *R*_merge_ (%)^b^ | 0.110 (–) | 0.066 (0.986) | 0.068 (0.740) |
| **Refinement** |  |  |  |
| Resolution limits (Å) | 42.8–3.0 (3.16–3.00) | 42.2–3.1 (3.28–3.09) | 41.8–3.1 (3.29–3.10) |
| Number of reflections in working set | 20,818 (2807) | 18,134 (2676) | 18,233 (2746) |
| Number of reflections in test set | 1061 (164) | 918 (150) | 926 (156) |
| *R*_xpct_ (%)^c^ | 0.1763 (0.2389) | 0.1784 (0.2541) | 0.1729 (0.2407) |
| *R*_free_ (%)^c,d^ | 0.1999 (0.2943) | 0.2122 (0.2979) | 0.1930 (0.2600) |
| Number of atoms | 5682 | 5703 | 5711 |
| Residues in Ramachandran favoured region (%) | 98.2 | 98.5 | 98.2 |
| Ramachandran outliers (%) | 0.0 | 0.0 | 0.0 |
| r.m.s.d bond lengths^e^ (Å) | 0.010 | 0.010 | 0.010 |
| r.m.s.d bond angles^e^ (°) | 1.02 | 1.01 | 1.04 |
| Average *B* factors (Å^2^) | 75.91 | 106.4 | 111.91 |

^a^Numbers in parentheses refer to the appropriate outer shell.

^b^*R*_merge_ = Σ_hkl_ Σ_i_|*I(hkl;i)* – <*I(hkl)*>|/Σ_hkl_ Σ_i_*I(hkl;i)*, where *I(hkl;i)* is the intensity of an individual measurement of a reflection and <*I(hkl)*> is the average intensity of that reflection. HKL2000 does not report *R*_merge_ values greater than 1.0.

^c^*R*_xpct_ = Σ_hkl_||*F*_obs_| –|*F*_xpct_||/Σ_hkl_ |*F*_obs_|, where |*F*_obs_| and |*F*_xpct_| are the observed structure facture amplitude and the expectation of the model structure factor amplitude, respectively

^d^*R*_free_ equals *R*_xpct_ of the test set (5% of the data removed prior to refinement).

^e^r.m.s.d. is root mean square deviation from ideal geometry
